# Myeloid Leukaemoid Reaction in a Surgical Case

**Published:** 1969-07

**Authors:** M. M. Ashraf


### MYELOID LEUKAEMOID REACTION IN A SURGICAL CASE

by

**M. M. Ashraf, M.B., B.S.**

### INTRODUCTION

The term " leukaemoid " was first used by Krumbhaar (1926) to describe changes in the peripheral blood suggesting leukaemia in patients suffering from conditions other than leukaemia. Many reports of leukaemoid reactions have appeared since then, and the subject has been reviewed by Heck and Hall (1939), Hill and Duncan (1941), and Hilts and Shaw (1953). A patient at Frenchay Hospital recently presented with an extremely high peripheral blood white cell count (reaching 132,000 per cmm.), with some immature cells, and it was thought that it would be useful to report this.

### CLINICAL FINDINGS

A farmer aged 65 years was admitted on 29.8.68 with a history of attacks of rigors and fever during the previous four weeks, followed by severe pain in the right loin, and subsequently by the passage of blood and small calculi in the urine. At this time he also developed pain in the back of his legs with swelling of the legs. He gave a history of attacks of passing blood-stained urine in the past, and he had lost some weight over the past year.

On examination, he was a well-built man with a blood pressure of 130/95. The relevant clinical findings included oedema of both legs and a palpable right kidney. There was some collapse of the right lung. X-ray examination confirmed the presence of a large stone in the right kidney, and intravenous pyelography failed to demonstrate any function in the right kidney. A barium meal revealed the presence of an ulcer crater on the posterior wall of the stomach and chest X-ray showed bilateral basal collapse. Blood findings are shown in Table I. The platelet count was normal.

By 14.9.68 some ascites and oedema of the abdominal wall had developed, with prominent superficial veins. A diagnosis of thrombosis of the inferior vena cava was considered. On 23.9.68 a right nephrectomy was performed. At operation, a large mass of necrotic material was encountered around the kidney, blending with the renal tissue. Histological examination showed gross chronic pyelonephritis, and widespread infiltration by a poorly differentiated transitional-cell carcinoma. The patient made a good recovery after the operation, and on 25.9.68 adult forms of *Ascaris lumbricoides* were found in the stools. He became confused, developed circulatory failure, his condition deteriorated rapidly, and he died three days later on 28.9.68.

On 10.9.68 a bone marrow aspiration had revealed the following:— promyelocytes 4% myelocytes 20% metamyelocytes 3%, stab cells 36%, neutrophils 22%, eosinophilic myelocytes 1%, adult eosinophils 1%, lymphocytes 2%, normoblast: basophilic 1%, polychromatic 8% orthochromic 1%. No excess of primitive cells was seen. Megakaryocytes were present in normal numbers. The myeloid cells were alkaline phosphatase positive and so were the granulocytes in the peripheral blood.

TABLE I

Peripheral Blood Findings

| Date | Hb (gm/100 ml.) | P.C.V. | W.B.C./cmm. | |
|---|---|---|---|---|
| 29.8.68 | 9.4 | 34% | 80,000 | |
| 9.9.68 | 8.5 | 28% | 132,000 | |
| 12.9.68 | 9.0 | 28% | 132,000 | (with " left shift " and toxic granulation) |
| 14.9.68 | 8.8 | 28% | 128,000 | |
| 17.9.68 | 9.0 | 29% | 110,000 | |
| 19.9.68 | 8.7 | 28% | 110,000 | (normal cell appearance) |
| 24.9.68 | 10.3 | 32% | 49,000 | (3% myelocytes) |
| 25.9.68 | 10.5 | 34% | 60,400 | (P=82%, Stab cells=15%) |
| 28.9.68 | 10.5 | 34% | 88,400 | ("left shift " only) |

In the interval of a month between his admission and his death, the patient's blood urea rose progressively from 54 to 209 mg/100 ml. On 10.9.68 the serum alkaline phosphatase was 357 I.U., the serum protein was 7.8 gm/100 ml., with globulin 5.1 gm./100 ml., and electrophoresis showed raised alpha-1 and alpha-2 globulins, and a marked increase in gamma globulin. There was no Bence-Jones protein in the urine.

**Post-mortem examiation** on 1.10.68 revealed a transitional-cell carcinoma of the right kidney, involving the renal vein and inferior vena cava; thrombosis of the inferior vena cava and left renal vein; pulmonary embolism and broncho-pneumonia.

### DISCUSSION

A leukaemoid reaction has been observed in some infections, intoxications, metastasizing tumours, and in some non-leukaemic blood diseases. Differentiation between leukaemoid reaction and leukaemia is not difficult, when the clinical findings and the results of examination of blood and bone marrow are considered together as shown in Table II.

TABLE II

| | Myeloid Leukaemoid Reaction | Myeloid Leukaemia |
|---|---|---|
| High peripheral blood white cell count | Yes | Yes |
| High peripheral blood " blast " cell count | No | Variable |
| Anaemia | If present, due to primary lesion | Frequent |
| Thrombocytopenia | Unusual | Frequent |
| Bone marrow | Non-leukaemic | Leukaemic |
| Neutrophil alkaline phosphatase | High score | Low score |

Three types of leukaemoid reaction have been described:—

1. Myeloid, in which the peripheral blood findings superficially resemble those of myeloid leukaemia.
2. Lymphoid, in which the peripheral blood findings suggest a diagnosis of lymphatic leukaemia. This type of reaction occurs in tuberculosis, malignancy, and in certain infective conditions such as whooping cough and infectious mononucleosis (Welsh *et al.* 1959).
3. Monocytic leukaemoid reaction. Only one case of this type has been reported. A 16 year old youth suffering from tuberculosis and a mediastinal teratoma developed a moncyctic leukaemoid reaction which was considered to be due to tuberculosis.

In this paper only myeloid leukaemoid reactions are considered, and these have been classified by Hill and Duncan (1941) as due to (1) bone marrow irritation or stimulation by physical, chemical, or allergic means, or (2) marrow response to overwhelming demand for release of leucocytes into the peripheral blood, or (3) ectopic haemopoiesis as a result of destruction of, or encroachment on, marrow space.

Myeloid leukaemoid reactions occur under the following conditions:—

1. **Bacterial infective conditions.** Leukaemoid reactions occur particularly in association with collections of pus in obscure sites such as the pelvis, the subphrenic region, following rupture of an encapsulated empyema into the uninfected pleural cavity (when a peripheral white blood count of 71,500 per cmm. was recorded, Smith, 1950), and in association with pneumonia (Stephens, 1934). Very high peripheral blood white cell counts of 76,800 per cmm. in pneumococcal endocarditis, 60,000 per cmm. in meningococcal meningitis, 70,000 per cmm. in diphtheria, 100,000 per cmm. in bubonic plague, and similar counts in septicaemia, due to staphylococci, streptococci, *Pseudomonas pyocyaneus* (reaching 112,000 per cmm.) have been recorded (Hilts and Shaw 1953). The reaction may also be found when purulent infections complicate Hodgkin's disease and related conditions. The reaction has been observed in congenital syphilis, and also in mixed infection due to malaria parasites (Riley and Robins, 1949).

2. **Non-leukaemic blood disorders.** Marked leucocytosis with a shift to the left in the nuclear lobe count has been observed in acute haemolysis and following severe acute haemorrhage, especially in young children (Holland Mover, 1963). This is a result of increased haemopoietic activity; for example in erythroblastosis foetalis there is release of immature myeloid cells as well as increased release of erythroid precursors from the bone marrow. Similarly the severe and sudden haemolysis of favism may cause a leukaemoid reaction.

3. **Intoxications.** Leukaemoid reactions as a result of irritation or abnormal stimulation of the bone marrow are rare, but they do occur in eclampsia (with recorded white cell counts up to 100,000 per cmm), acute hepatic failure, severe burns (up to 80,000 per cmm, Krumbhaar 1926), mustard gas and mercucy poisoning (69,500 white cells per cmm., Downey, Major and Noble, 1930). They may also occur during recovery from drug-induced depression. During the treatment of malignancy with either irradiation or chemotherapy a marked leucopenia may occur as a result of induced bone marrow hypoplasia, and this may be followed by a regenerative phase causing release of immature myeloid cells from the marrow producing a myeloid leukaemoid reaction. Leukaemoid reactions have been observed in rheumatoid arthritis, and in Addison's and other megaloblastic anaemias (Callendar 1944). They have occasionally been noticed after treatment with folic acid and liver extract (Sclare and Cragg, 1958).

4. **Malignancy and disseminated malignant metastases.** Leukaemoid reactions have been observed as a result of bone marrow invasion by tumour cells from primary tumours in the lungs, stomach, breast, kidneys, prostate, testis, and malignant melanoma (Meyer and Rotter 1942, Zarofnetis and Joseph 1961). Leukaemoid reactions can occur in malignant diseases without invasion of the bone marrow, and the mode of production of the reaction is not understood. Moore and Pickren (1967) have observed ultra-microscopic particles resembling herpes virus in cultures of haemopoietic cells derived from lymphoma, malignant melanoma, non-haemopoietic malignancies, and in various leukaemias. They have also reported a similar finding in a patient who returned with metastases 2 years after surgical removal of a malignant melanoma. Tissue culture revealed the presence of a " leucovirus ". A homologous tumour tissue transplant was performed, and the bone marrow revealed an eosinophilic leukaemoid reaction, but no tumour cells or other abnormal cells were seen in the marrow. It has therefore been postulated that the occurrence of a leukaemoid reaction to malignancy or to haemopoietic toxins could be due to the action of the " leucovirus ", or alternatively malignancy could lower the body's defence mechanisms sufficiently to allow the " leucovirus ' to multiply.

5. **Non-reactive tuberculosis.** Leukaemoid reactions occurring in non-reactive tuberculosis were first observed by Roth (1913) and Marshal (1915), and since then many similar cases have been described in which the blood and bone marrow examination revaled apparent leukaemia, but in which later autopsy revealed miliary tuberculous infiltration but no evidence of leukaemia (Custer and Crocker, 1963; Withers, 1964).

**Acknowledgements.** I am grateful to Dr. R. D. Eastham, Consultant Pathologist, for his helpful advice, guidance and encouragement. I am also indebted to Dr. R. J. Sandry, Consultant Pathologist, and Mr. Davidson., Consultant Surgeon, for helping me to follow this case. I also thank Mr. E. Morgan, Mr. K. Dance, Miss Patricia Walker, Mrs. J. Cross, and Miss D. Woods for their skilled technical assistance.

REFERENCES

Custer, R. P., and Crocker, W. J. (1932) Folia haemat. (Leipzig) **46:** 359.

Downey, H., Major, S. G., and Noble J. F. (1930). Folia haemat. **41,** 493.

Callendar, S. T. E. (1944). Quart J. Med. **13,** 75.

Hill, J. M., and Duncan, C. N. (1941) Amer. J. med. Sci. **201:** 847.

Hilts, S. V., and Shaw, C. C. (1953) New England Journal of Medicine, **249,** 434.

Heck, F. J., and Hall, B. E. (1939) J.A.M.A. **23,** 95.

Holland, D. P., Mover, A. M. (1963) American Journal Diseases of Children, **105,** 568.

Krumbhaar, E. B. (1926) Amer. J. med. Sci. **172,** 519.
Meyer, L. M., and Rotter, S. D. (1942) American Journal of Clinical Pathol-ogy, **12,** 218.
Moore, G. E., Pickren, J. W. (1967). Laboratory Investigation **16,** 882.
Riley, J. S. and Robins, G. M. (1949) Blood **4,** 283.
Sclare, G., and Cragg, J. A. (1958) Journal Clinical Pathology, **11**, 45.
Stephens, D. J. (1934) American Journal of Medicine Sci. **188;** 332.
Smith, M. D. (1950) Glasgow Medical Journal **31,** 82.
Welsh, J. D., Denny, W. F. and Bird, R. M. (1959) Southern Medical Journal **52,** 643.
Withers, K. L. (1964) Medical Journal of Australia, **2,** 242.
Zarafonets, C. J. D., and Joseph, Rosaline R. (1961) American Journal of Medical Sci. **240,** 750.

